# The alteration of intestinal mucosal α-synuclein expression and mucosal microbiota in Parkinson’s disease

**DOI:** 10.1007/s00253-023-12410-w

**Published:** 2023-02-16

**Authors:** Jihua Shi, Yiran Wang, Dan Chen, Xue Xu, Wenbin Li, Kai Li, Jing He, Wen Su, Qingfeng Luo

**Affiliations:** 1 Department of Gastroenterology, Beijing Hospital, National Center of Gerontology, Institute of Geriatric Medicine, Chinese Academy of Medical Science, No. 1 DaHua Road, DongDan, Beijing, 100730 China; 2 Peking University Fifth School of Clinical Medicine, Beijing, China; 3 Department of Neurology, Beijing Hospital, National Center of Gerontology, Institute of Geriatric Medicine, Chinese Academy of Medical Science, Beijing, China

**Keywords:** Parkinson’s disease, α-Synuclein, Mucosal microbiota, Intestinal mucosa, Diagnosis

## Abstract

**Abstract:**

Parkinson’s disease (PD) is the second most common neurodegenerative disease but still lacks a preclinical strategy to identify it. The diagnostic value of intestinal mucosal α-synuclein (αSyn) in PD has not drawn a uniform conclusion. The relationship between the alteration of intestinal mucosal αSyn expression and mucosal microbiota is unclear. Nineteen PD patients and twenty-two healthy controls were enrolled in our study from whom were collected, using gastrointestinal endoscopes, duodenal and sigmoid mucosal samples for biopsy. Multiplex immunohistochemistry was performed to detect total, phosphorylate, and oligomer α-synuclein. Next-generation 16S rRNA amplicon sequencing was applied for taxonomic analysis. The results implied that oligomer α-synuclein (OSyn) in sigmoid mucosa of PD patients was transferred from the intestinal epithelial cell membrane to the cytoplasm, acinar lumen, and stroma. Its distribution feature was significantly different between the two groups, especially the ratio of OSyn/αSyn. The microbiota composition in mucosa also differed. The relative abundances of *Kiloniellales*, *Flavobacteriaceae*, and *CAG56* were lower, while those of *Proteobacteria*, *Gammaproteobacteria*, *Burkholderiales*, *Burkholdriaceae*, *Oxalobacteraceae*, *Ralstonia*, *Massilla*, and *Lactoccus* were higher in duodenal mucosa of PD patients. The relative abundances of *Thermoactinomycetales* and *Thermoactinomycetaceae* were lower, while those of *Prevotellaceae* and *Bifidobacterium longum* were higher in patients’ sigmoid mucosa. Further, the OSyn/αSyn level was positively correlated with the relative abundances of *Proteobacteria*, *Gammaproteobacteria*, *Burkholderiales*, *Pseudomonadales*, *Burkholderiaceae*, and *Ralstonia* in the duodenal mucosa, while it was negatively correlated with the Chao1 index and observed operational taxonomic units of microbiota in sigmoid mucosa. The intestinal mucosal microbiota composition of PD patients altered with the relative abundances of proinflammatory bacteria in the duodenal mucosa increased. The ratio of the OSyn/αSyn level in the sigmoid mucosa indicated a potential diagnostic value for PD, which also correlated with mucosal microbiota diversity and composition.

**Key points:**

*• The distribution of OSyn in sigmoid mucosa differed between PD patients and healthy controls.*

*• Significant alterations in the microbiome were found in PD patients’ gut mucosa.*

*• OSyn/αSyn level in sigmoid mucosa indicated a potential diagnostic value for PD.*

**Supplementary Information:**

The online version contains supplementary material available at 10.1007/s00253-023-12410-w.

## Introduction

Parkinson’s disease (PD) is the second most common neurodegenerative disease after Alzheimer’s disease (Tysnes et al. 2017). In PD patients, disruption of the α-synuclein (αSyn) structure biases it toward aggregation-prone conformations (Stephens et al. 2019). This accumulation is not limited to the central nervous system but is also found in the enteric nervous system in the postmortem (Ruffmann et al. 2016), making αSyn detection via gastrointestinal biopsies a hot research topic for PD diagnosis. Several studies suggest that αSyn pathology might begin and be detectable in the gastrointestinal tract during the prodromal PD phase before affecting the brain (Hilton et al. 2014; Stokholm et al. 2016). In addition, non-motor symptoms of PD, especially in the gastrointestinal tract, could appear 10 years earlier than motor symptoms, resulting in PD patients with long-term severe constipation (Holmqvist et al. 2014).

Historically, the diagnosis of PD has relied on clinical symptoms and can only be confirmed by postmortem examination of misfolded αSyn in the brain with neuronal loss in the substantia nigra (Dickson et al. 2009), which lacks a preclinical strategy to help identify PD by in vivo biomarkers. Given the close relationship between intestinal αSyn and PD, we hypothesized that the detection of intestinal mucosal αSyn expression would be helpful for the early diagnosis of PD. However, in recent years, αSyn has also been found in healthy people (Coker et al. 2018; Ruffmann et al. 2018), leading to the difficulty of distinguishing PD from normal controls. An assumption is that the “non-PD controls” in which αSyn deposition is detected are also PD patients, but no follow-up study is available to verify and diagnose their disease status. Besides, the previous studies that mainly detected the total and phosphorylate αSyn (PSyn) in the intestinal mucosa showed contradictory conclusions on the reliability of those indicators.

Some recent research reported that the oligomer αSyn (OSyn) was toxic to the nervous system and may relate to the pathogenesis (Ludtmann et al. 2018), while no one has examined its expression in the gastrointestinal mucosa. Intestinal mucosal αSyn might have a potential diagnostic value for PD, but the methodology used in the existing studies cannot make it a gold standard. Hence, one aim of this study is to use multiple fluorescence immunohistochemical technology to simultaneously detect three different αSyn species including the total, phosphorylate, and oligomer types to explore the potential PD biomarkers by intestinal mucosal biopsy. Besides, dysfunction of the microecology-gut-brain axis plays an important role in the pathogenesis of PD. However, previous studies about microecological disorders of PD are mainly based on fecal microbiota, and there is a lack of understanding of the intestinal mucosal microbiota in PD patients. A few studies have reported the characteristics of colonic mucosal microbiota in PD patients, but there are no related reports on the characteristics of the upper gastrointestinal mucosal flora in PD patients. Thus, the other aim of this study is to study the characteristics of duodenal mucosal and sigmoid mucosal microbiota in PD patients.

## Materials and methods

### Study design

The patients with severe constipation and who were willing to undergo gastrointestinal endoscopy were recruited in our cohort. For the PD group, the volunteers needed to be diagnosed by an experienced neurologist (the stage of Hoehn and Yahr Scale < 5) (Hoehn et al. 1967). We enrolled counterpart-age patients without any other neurodegenerative diseases for the control group. This study was conducted under the ethical principles of the Declaration of Helsinki. The study protocol was approved by Beijing Hospital with the approval number 2019BJYYEC-199-03. Written informed consent was obtained from all subjects before participation. All subjects were informed of the potential risk of bleeding during and after the biopsy.

### Collection of biopsy specimens

Biopsy specimens of the duodenal mucosa and sigmoid mucosa were obtained during gastroscopy and colonoscopy. Some of the specimens were preserved in formalin, and 4-μm-thick paraffin sections were prepared for further staining. The other obtained fresh mucosal samples were frozen at − 80 °C within 1 h before subsequent mucosal flora sequencing analysis. Any biopsy that consists entirely or predominantly of abnormal tissue (e.g., carcinoma) was excluded from the final calculation.

### Multiplex immunohistochemistry

Multiplex immunohistochemistry (mIHC) was performed according to a sequential multiplexed immunofluorescence protocol (Stack et al. 2014). Briefly, total αSyn (Cat 1B10E9, Proteintech, Chicago, IL, USA), PSyn (Cat MABN826, Merck-Millipore, Boston, MA, USA), and OSyn (Cat ABN2265, Merck-Millipore) were co-stained with corresponding antibodies on the same sample. Then, corresponding secondary antibodies were used in fluorescein isothiocyanate–tyramide signal amplification (FITC-TSA) (PPD520, Panovue, Beijing, China) for total αSyn detection, CY3-TSA (PPD570, Panovue, Beijing, China) for PSyn detection, and CY5-TSA (PPD620, Panovue, Beijing, China) for OSyn detection. Nuclei were highlighted by DAPI (4′,6-diamidino-2-phenylindole) (D9542, Sigma-Aldrich, St Louis, USA). Image J software (Version 1.8.0.112; National Institutes of Health, Bethesda, USA) was applied for location determination and fluorescent intensity evaluation.

### Intestinal mucosal bacterial DNA extraction

Bacterial DNA was extracted from the mucosal samples at Novogene Bioinformatics Technology Co. Ltd. (Beijing, China) using the sodium dodecyl sulfate (SDS) method (Natarajan et al. 2016). DNA concentration and purity were monitored on agarose gels. After the concentration was determined, DNA was diluted to 1 ng/μL using sterile water.

### 16S rRNA gene sequence sequencing

16S rRNA genes of the 16S V4 region were amplified using specific primers (Caporaso et al. 2011) (515F: GTGCCAGCMGCCGCGGTAA; 806R: GGACTACHVGGGTWTCTAAT) with a barcode. The purity and concentration of DNA were detected by 2% agarose gel electrophoresis. Then, the target bands (400–450 bp) were purified and recovered for further experiments. Sequencing libraries were generated using an Illumina TruSeq DNA PCR-Free Library Preparation Kit (Illumina, San Diego, CA, USA) following the manufacturer’s recommendations. The library quality was assessed on the Qubit@ 2.0 Fluorometer (Thermo Fisher Scientific, Waltham, MA, USA). The library was sequenced on the NovaSeq6000 platform. Sequence data were deposited to the Sequence Read Archive (https://www.ncbi. nlm. nih. gov/ sra/) in the bioproject of PRJNA881840.

### 16S rRNA gene sequence analysis

(1) Amplicon sequence variants (ASVs): Classify-sklearn algorithm of QIIME2 (Bolyen et al. 2019) used to annotate species for each ASV was used a pre-trained naϊve Bayes classifier. (2) Biodiversity coverage: The biodiversity coverage can directly reflect the rationality of the amount of sequencing data and indirectly reflect the richness of species in the samples. We depicted the rarefaction curves using QIIME2 to show the calculation of species richness and to reflect the biodiversity coverage (Boussarie et al. 2018). When the curve tends to be flat, it shows that the amount of sequencing data is enough, and more data will only produce a small number of new species. (3) Alpha diversity: Alpha diversity was applied in analyzing the complexity of species diversity for a sample through 4 indices including observed operational taxonomic units (OTUs), Chao1, Shannon, and Simpson. We used QIIME2 to achieve this. (4) Beta diversity: Beta diversity analysis was used to evaluate differences of samples in species complexity. Principal coordinate analysis (PCoA) was performed to get principal coordinates and visualize from complex, multidimensional data. Samples with high similarity in community structure tend to gather together, and samples with large community differences will be far away. Analysis of similarities (ANOSIM), calculated by QIIME2, was used to test whether the difference between groups was significantly greater than that within groups. The *R* statistic, a ratio between within-group and between-group dissimilarities, was between (− 1, 1). *R* greater than 0 indicated that the difference between groups was significant. *R* less than 0 indicated that the difference within the group was greater than the difference between groups. (5) Columnar accumulation diagram: A columnar accumulation diagram of the relative abundance of the top 10 species at the phylum and genus levels was used to show the global composition of the bacteria of each group. The plots were drawn by R (version 2.15.3), applying packages Vegan and Reshape. (6) Linear discriminant analysis effect size (LEfSe): LEfSe conducted by LEfSe software (Version 1.0) (Segata et al. 2011) was used to determine which species differed significantly between groups, and the threshold of the linear discriminant analysis (LDA) score was set to 4. (7) Spearman correlation analysis was used to analyze the correlation between mucosal αSyn expression level and alpha diversity and the relative abundance of the top 20 species at phylum, class, order, family, and genus levels of mucosal flora. (8) Functional prediction: Functional prediction was carried out based on KEGG databases (https://www.genome.jp/kegg/). The top 35 functions in abundance were selected to draw a heat map.

### Statistical analysis

Statistical analysis was performed by SPSS 26.0 (SPSS Inc., Chicago, IL, USA). The age of our cohort is nonnormal data and is thus being expressed as average (minimum, maximum) deviation using the Wilcoxon test. Counting data were compared using the chi-square test. *P* value < 0.05 was considered statistically significant in all tests. The mean fluorescence intensity was calculated by Image J (Version 1.8.0.112; National Institutes of Health, Bethesda, USA). The scatterplots and column diagram were exhibited by GraphPad Prism 9.0 (GraphPad Software Inc., San Diego, CA, USA), and the receiver operation characteristic (ROC) curves were depicted by using the web-tool easyROC (http://www.biosoft.hacettepe.edu.tr/easyROC/). The closer the value of the area under curve (AUC) is to 1, the better the diagnostic efficiency is.

## Results

### Clinical characteristics of enrolled subjects

Nineteen patients and 22 controls were recruited in our cohort to undergo gastrointestinal endoscopy and tissue biopsy. One of the PD patients who had multiple colon polyp forceps provided one biopsy for our study. One of the controls was only acquired from duodenum biopsy due to inadequate preparation for colon cleansing. The basic information of the final enrolled samples in the PD and control groups is shown in Table 1.

**Table 1:** Basic information of enrolled subjects

		PD	Control	*P* value^a^
Number of subjects		19	22	
Gender	Male	12	16	0.511
	Female	7	6	
Age^b^		65 (61, 75)	64 (56, 68)	0.129
Location	Sigmoid	20	21	0.570
	Duodenum	10	7	

^a^*P* values were calculated using the chi-square test (gender and location) and Wilcoxon test (age) to evaluate the characteristics between the two groups. *P* value > 0.05 was considered no statistical difference

^b^Exhibiting as average (minimum, maximum)

In the present study, duodenal mucosal specimens from 7 healthy controls and 10 PD patients and sigmoid mucosal specimens from 21 healthy controls and 19 PD patients were used to explore the expression of αSyn by mIHC. After the quality control filtration of the amplicon, duodenal mucosal specimens from 7 healthy controls and 7 PD patients and sigmoid mucosal specimens from 18 healthy controls and 17 PD patients were analyzed on the Illumina NovaSeq sequencing platform. The double-end sequencing method of the V4 region of 16S rRNA was performed for the intestinal mucosal microbiota.

### The distribution of different types of αSyn in the intestinal mucosa was different between the PD and control subjects

**Fig. 1:**
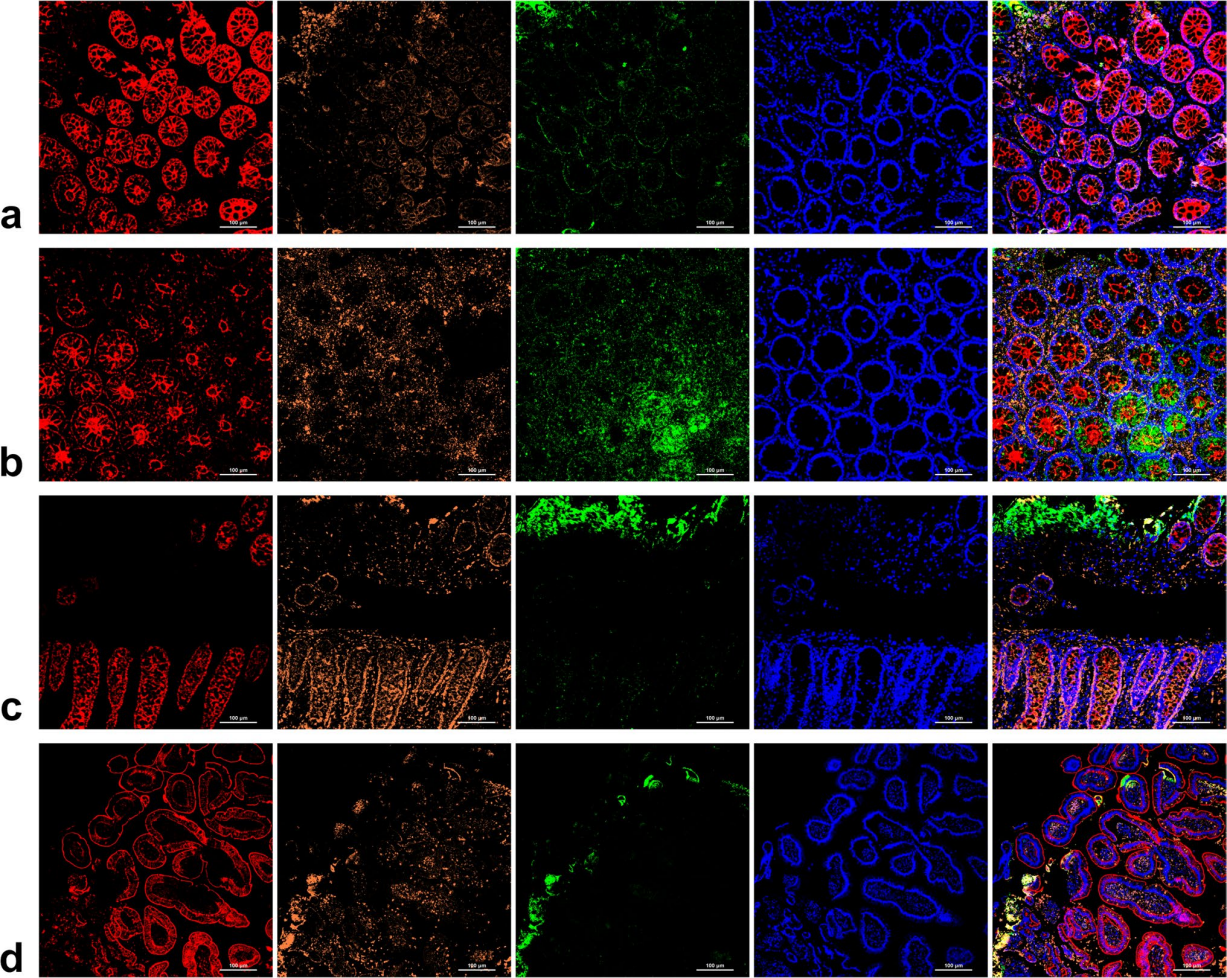
The distribution of different types of α-synuclein in the sigmoid mucosa of **a** normal controls and **b**–**d** PD patients; ×200. The scale bars are in the bottom right corner of each picture, indicating 100 μm. Total αSyn staining, red; OSyn staining, orange; PSyn staining, green; DAPI staining, blue; PD: Parkinson’s disease; αSyn: α-synuclein; OSyn: oligomer α-synuclein; PSyn: phosphorylate α-synuclein

By applying mIHC to detect the three types of αSyn, the landscape of them in intestinal mucosa was depicted (Fig. 1). As observed, in normal controls, αSyn was mainly distributed in the intestinal epithelial cell membrane and a little in the cytoplasm (Fig. 1a). In PD patients, αSyn was distributed in the intestinal epithelial cell membrane and stroma (Fig. 1b–d). The OSyn aggregated from αSyn (Castillo-Carranza et al. 2018) was mainly distributed in the intestinal epithelial cell membrane in normal controls (Fig. 1a), while in PD patients, it was mainly distributed in the stroma and acinar lumen with only a small amount in the intestinal epithelial cell cytoplasm (Fig. 1b–d). As to PSyn, in normal controls, it was also mainly distributed in the intestinal epithelial membrane (Fig. 1a), while in PD patients, PSyn was mainly distributed in the intestinal epithelial membrane and cytoplasm (Fig. 1b–d). To sum up, the distribution of the three types of αSyn in colon mucosae of PD patients differed from the control ones. In particular, OSyn was the most prominent. In the sigmoid mucosa biopsy specimens from PD patients, OSyn was transferred from the intestinal epithelial cell membrane to the cytoplasm, acinar lumen, and stroma. However, the descriptions above were more in accord with that colon tissue rather than in the duodenum samples (Supplemental Fig. S1).

### The fluorescence intensity ratio of different types of αSyn in colonic mucosa showed a valid diagnosis of PD disease

**Fig. 2:**
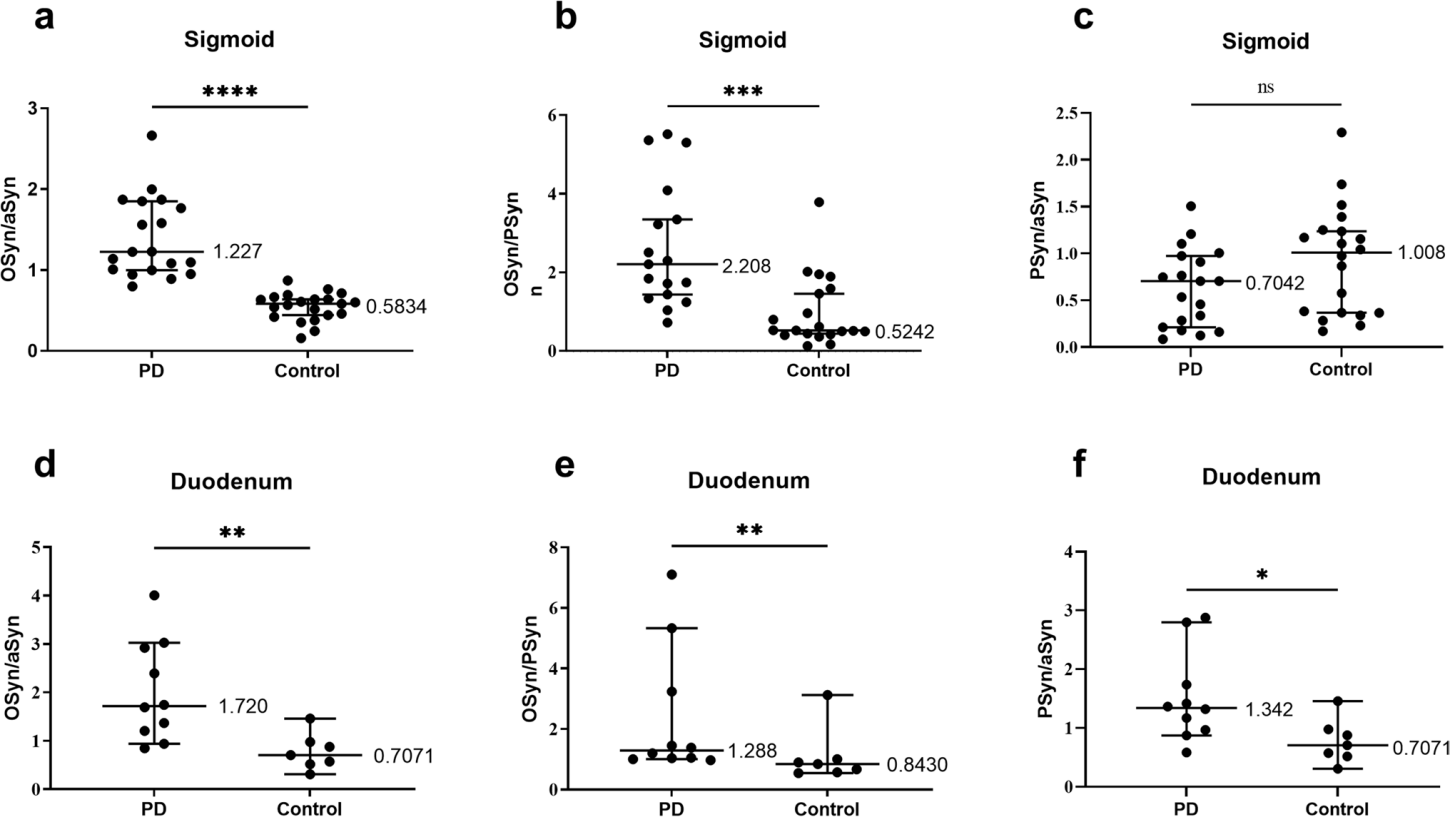
Comparison of the mean fluorescence intensity ratio of different types of α-synuclein in sigmoid. **a** OSyn/αSyn in sigmoid mucosa; **b** OSyn/PSyn in sigmoid mucosa; **c** PSyn/αSyn in sigmoid mucosa; **d** OSyn/αSyn in duodenum mucosa; **e** OSyn/PSyn in duodenum mucosa; **f** PSyn/αSyn in duodenum mucosa. *n* = 7–21; ns *P* > 0.05, *P < 0.05, **P < 0.01, ***P < 0.001,****P<0.0001. PD: Parkinson’s disease; OSyn/αSyn: oligomer α-synuclein/total α-synuclein; OSyn/PSyn: oligomer α-synuclein/phosphorylate α-synuclein; PSyn/αSyn: phosphorylate α-synuclein/total α-synuclein

**Fig. 3:**
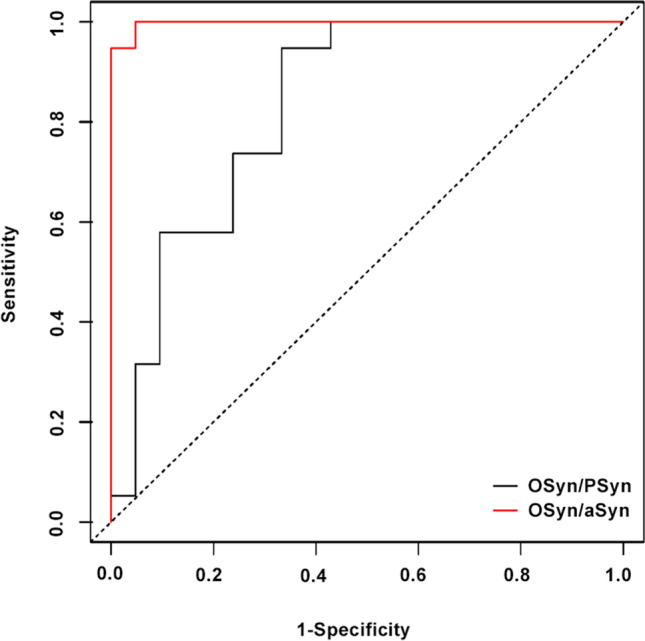
ROC analysis values of the ratio of different αSyn types in PD diagnosis. Black line: OSyn/PSyn; red line: OSyn/αSyn; OSyn/αSyn: oligomer α-synuclein/total α-synuclein; OSyn/PSyn: oligomer α-synuclein/phosphorylate α-synuclein

Based on the observations of αSyn distribution, we quantified the mean fluorescence intensity of each channel. Considering the systematic errors between experimental batches, we calculated the ratios between diverse αSyn (Fig. 2). From the data, oligomer α-synuclein/total α-synuclein (OSyn/αSyn) and oligomer α-synuclein/phosphorylate α-synuclein (OSyn/PSyn) of the sigmoid colon demonstrated significant differences between the PD and control groups. Furthermore, we calculated the ROC curve to evaluate the diagnostic efficiency of each ratio (Fig. 3) and the ROC statistics are displayed in Table 2. The ratio of OSyn/αSyn indicates an effective ability to distinguish PD patients and controls, with the cut-off point of 0.799.

**Table 2:** The related statistics data of receiver operation characteristic (ROC) curves

Ratio	AUC	SE AUC	Lower limit	Upper limit	*P* value
OSyn/αSyn	0.99749	0.00354	0.99055	1.00444	0
OSyn/PSyn	0.83208	0.06621	0.70231	0.96185	0

*P* value < 0.05 implied the ratios were statistically different between the PD and control groups

*AUC* area under curve, *SE AUC* standard error of AUC

### The relationship between other factors and the different types of αSyn expression levels in sigmoid mucosa

To validate the diagnostic efficiency, we evaluated other factors that possibly influence the expressions of all types of αSyn. Supplemental Fig. S2 showed that the expression level of different types of αSyn in sigmoid mucosa exhibited no difference between men and women. Our data (Supplemental Fig. S3) did not show the statistical correlations between age and any types of αSyn.

### Mucosal microbiome ASVs

An average of 105,232 and 105,918 pyrosequencing reads per sample of duodenal mucosa and sigmoid mucosa were generated after sequencing, respectively. After proper quality control, the number of reads per sample was reduced to 69,114 and 70,791, respectively. The level of biodiversity coverage was assayed through observed species number biodiversity curves (Supplemental Fig. S4), which tended to plateau for each sample, confirming the high accuracy of the performed 16S rRNA profiling analysis.

### Comparison of the mucosal microbiota intra- and inter-individual variability among the different groups

**Fig. 4:**
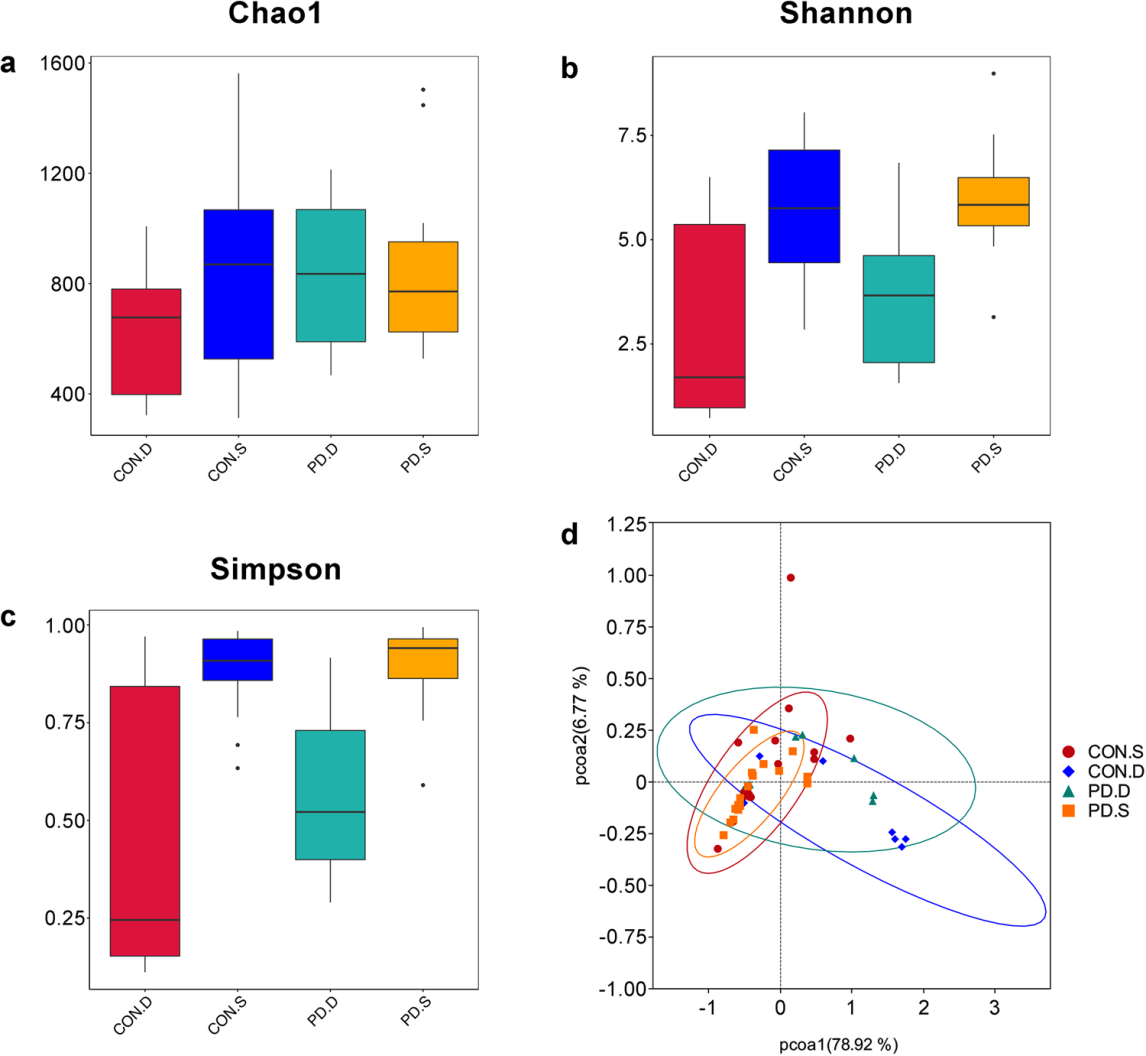
Alpha and beta diversity analysis of duodenal and sigmoid mucosal microbiota. **a** Chao1 richness estimator, **b** Shannon biodiversity index, **c** Simpson diversity index, **d** unweighted-unifrac principal coordinate analysis (PCA)

The alpha diversity assessed by three different indexes including Chao1 richness estimator (Fig. 4a), Shannon biodiversity index (Fig. 4b), and Simpson diversity index (Fig. 4c) were used to analyze the complexity of species diversity. Compared to the duodenal mucosa of controls or PD patients, Kruskal–Wallis analysis showed that the microbiotic diversity of sigmoid mucosa was significantly richer according to the Shannon and Simpson diversity indices (*P* < 0.05). There was no significant difference in the diversity of the duodenal or sigmoid mucosa between the controls and PD patients according to the Chao1, Shannon, and Simpson diversity indices (*P* > 0.05). The beta diversity among the samples allowed a detailed analysis of the similarities between the gut microbiota composition of the different groups. PCoA (Fig. 4d) revealed that the samples of duodenal mucosa and sigmoid mucosa from PD patients clustered separately (*R* = 0.30, *P* = 0.030), while the duodenal or sigmoid mucosal samples of the controls and PD patients appeared to cluster together (*R* > 0), which was confirmed by ANOSIM.

### Taxonomic analysis of mucosal microbiota composition among different groups

**Fig. 5:**
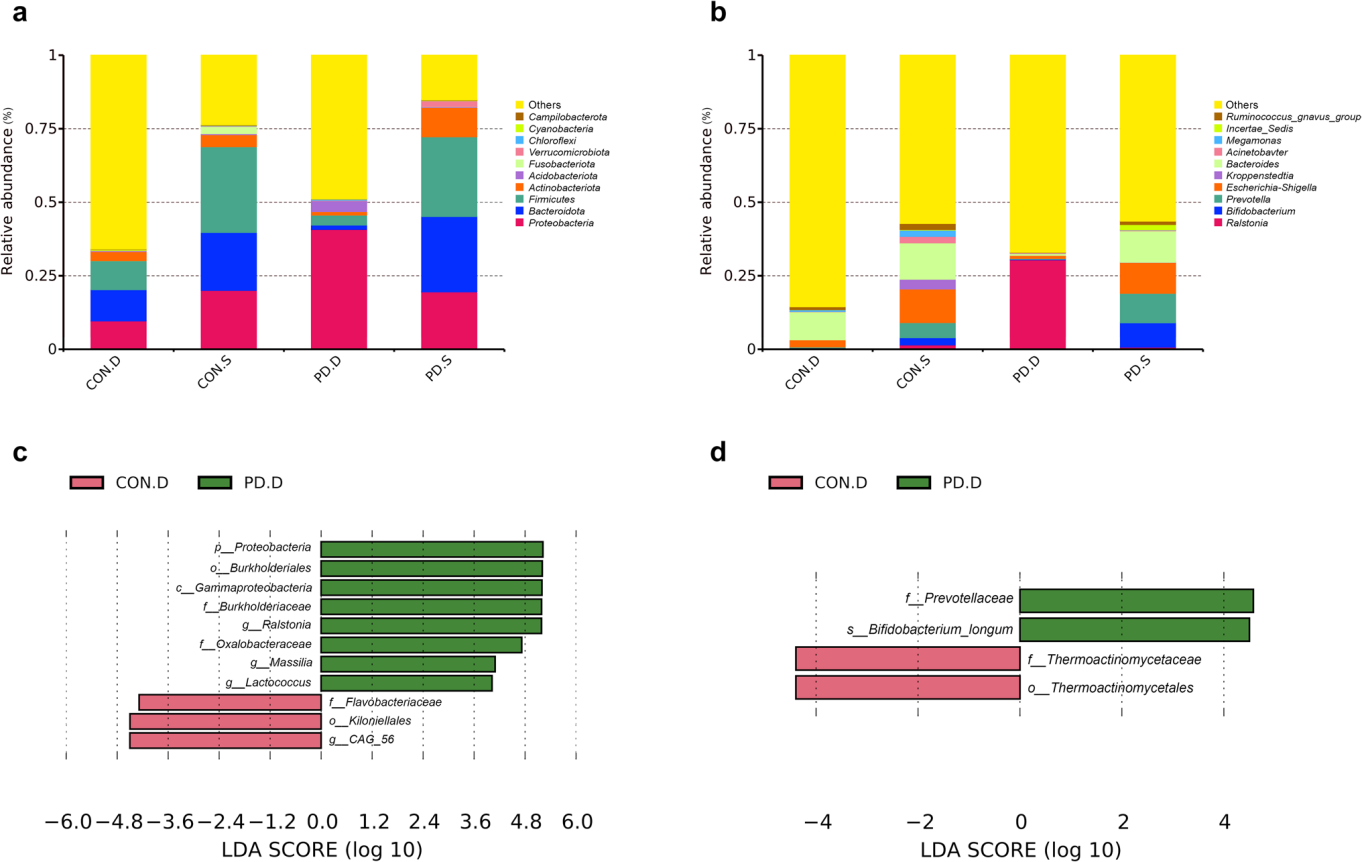
Duodenal and sigmoid mucosal microbiota composition and differential species analysis. Columnar accumulation diagram of relative abundances of the top 10 species at the phylum (**a**) and genus (**b**) levels. Linear discriminant analysis effect size of duodenal (**c**) and sigmoid (**d**) mucosal microbiota

The composition of the top 10 species at the phylum and genus levels was analyzed by the column accumulation diagram of relative abundances of species. At the phylum level (Fig. 5a), the relative abundances of *Bacteroidetes* and *Proteobacteria* were the highest in the duodenal mucosa of the controls and PD patients, respectively, while those of *Firmicutes* in the sigmoid mucosa of the controls and PD patients were the highest. At the genus level (Fig. 5b), *Bacteroides* and *Ralstonia* had the highest relative abundance in the duodenal mucosa of the controls and PD patients, respectively, while in the sigmoid mucosa of both the controls and PD patients, *Bacteroides* had the highest relative abundance.

To identify more precisely, we directly compared the bacterial composition at the phylum, class, order, family, genus, and species levels of the controls and PD patients from the results of the V4 hypervariable region of 16S rRNA gene sequencing, using LEfSe analysis. The results showed that in terms of duodenal mucosal microbiota (Fig. 5c), the relative abundances of *Kiloniellales* (at order the level), *Flavorbacteriaceae* (at the family level), and *CAG56* (at the genus level) in the control group were higher than those in the PD group. The relative abundances of *Proteobacteria* (at the phylum level), *Gammaproteobacteria* (at the class level), *Burkholderiales* (at the order level), *Burkholderiaceae* (at the family level), *Oxalobacteraceae* (at the family level), *Ralstonia* (at the genus level), *Massilla* (at the genus level), and *Lactococcus* (at the genus level) in the normal control group were lower than those in the PD group. In terms of sigmoid mucosal microbiota (Fig. 5d), the relative abundances of *Thermoactinomycetales* (at the order level) and *Thermoactinomycetaceae* (at the family level) in the control group were higher than those in the PD group. The relative abundances of *Prevotellaceae* (at the family level) and *Bifidobacterium longum* (at the species level) in the control group were lower than those in the PD group.

### Correlation analysis between mucosal OSyn/ αSyn expression level and mucosal microbiota composition

**Fig. 6:**
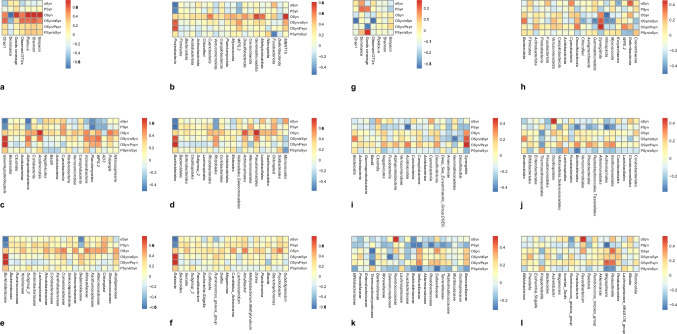
Correlation analysis of mucosal αSyn expression with alpha diversity and species abundances of mucosal microbiota. Spearman correlation analysis of duodenal mucosal αSyn expression with alpha diversity (**a**) and species abundances of duodenal mucosal microbiota at phylum (**b**), class (**c**), order (**d**), family (**e**), and genus (**f**) levels. Spearman correlation analysis of sigmoid mucosal αSyn expression with alpha diversity (**g**) and species abundances of sigmoid mucosal microbiota at phylum (**h**), class (**i**), order (**j**), family (**k**), and genus (**l**) levels

Spearman correlation analysis (Fig. 6a–f) showed that OSyn/ αSyn in duodenal mucosa was positively correlated with the relative abundance of *Proteobacteria* at the phylum level, *Gammaproteobacteria* at the class level, *Burkholderiales* and *Pseudomonadales* at the order level, *Burkholderiaceae* at the family level, and *Ralstonia* at the genus level.

OSyn/ αSyn in sigmoid mucosa was negatively correlated with the Chao1 index and observed OTUs of sigmoid mucosa microbiota (Fig. 6g). Besides, OSyn/ αSyn in the sigmoid mucosa was negatively correlated with the relative abundances of *Chloroflexi*, *Gemmatimonadota*, *Nitrospirota*, and *Myxococota* at the phylum level. In addition, OSyn/ αSyn in the sigmoid mucosa was negatively correlated with the relative abundance of *Gemmatimonadetes* and positively correlated with the relative abundance of *Synergia* at the class level (Fig. 6h–l).

### Functional prediction

**Fig. 7:**
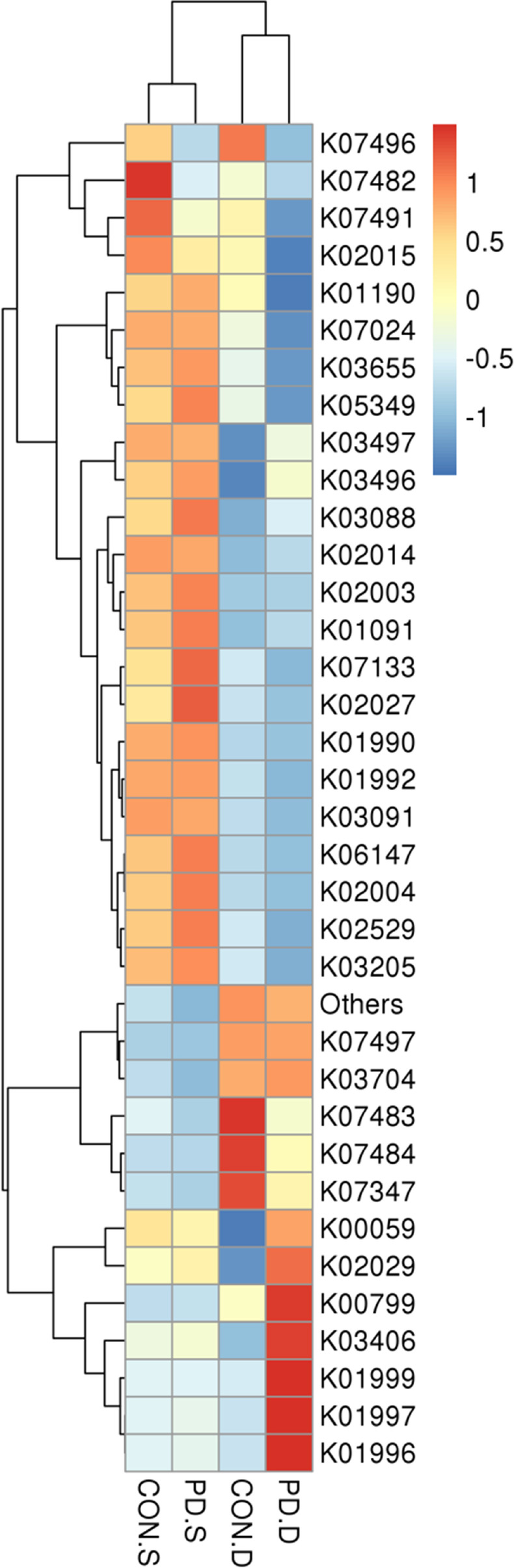
Functional profile prediction among different groups. A heat map of the top 35 functions in abundance based on KEGG database

Based on the KEGG method (Fig. 7), pathways correlated with glutathione S-transferase (K00799), methyl acceding chemotaxis protein (K03406), branched-chain amino acid transport system substrate-binding protein (K01999), branched-chain amino acid transport system permease protein (K01997), and branched-chain amino acid transport system ATP-binding protein (K01996) were enriched in the duodenal mucosa of PD patients, while the pathways correlated with putative ABC transport system ATP-binding protein (K02003), phosphoglycolate phosphatase (K01091), and multiple sugar transport system substrate-binding proteins (K02027) were enriched in the sigmoid mucosa of PD patients.

## Discussion

At present, it is believed that the role of the microecology-gut-brain axis in the pathogenesis of PD is as follows: imbalance of intestinal microecology and its metabolites leads to intestinal inflammation and intestinal mucosal barrier impairment. Then, endotoxin and pathogen invasion promote intestinal inflammation, leading to oxidative stress damage and misfolding of αSyn, which activates microglia and further aggravates inflammation. The accumulated αSyn in the gastrointestinal tract is transmitted to the central nervous system through the vagus nerve, resulting in oxidative stress and inflammation. As gastroenterologists, we keep an eye on the gastrointestinal manifestations of PD, comprising dysphagia, delayed gastric emptying, anorectal dysfunction, and especially severe constipation. Hence, our study aimed to analyze the expression of the three different αSyn species by mIHC, including the total, phosphorylate, and oligomer types. In our study, the distribution features of those proteins were different between the PD patients and controls. In addition, the ratios of OSyn/αSyn and OSyn /PSyn in the PD patients were significantly higher than those in the controls. The further ROC analysis of those ratios exhibited that OSyn /αSyn had a potential diagnosis efficiency.

As shown in our study, the total αSyn existed in both groups with different distributions in the intestinal mucosa. Our results indicated that OSyn was transferred from the intestinal epithelial cell membrane to the cytoplasm, acinar lumen, and stroma. OSyn was excreted from the mucous layer, which might partly support the assumption that enteroendocrine cells lining the intestinal tract serve as a reservoir for the central spread of the misfolded αSyn (Chandra et al. 2017) and the transport mediators between cells are exosomes (Guo et al. 2020). Considering that the the OSyn is a pathologic aggregation form of αSyn, the increasing portion of OSyn within the Syn pool also implied the overproduction of OSyn from the gut may be related to the pathological condition, which conformed to the toxic protein accumulation and gut-brain-axis retrograde transport theory (Travagli et al. 2020). Thus, an OSyn/αSyn ratio shows the potential of being a biomarker for PD diagnosis.

Furthermore, we analyzed whether the values and ratios of αSyn species would be influenced by gender or age and no correlation was found. Yang et al. (2020) have shown that the expression of αSyn and OSyn increases normally in the striatum and hippocampus, and Bottner et al. (2012) proposed that the presence of PSyn in the enteric nervous system (ENS) was age-related and might not necessarily be pathological. Taking all the results together, it is possible that the αSyn was formulated in the enteroendocrine cell. The OSyn emerged with aging and retrograde transport to the brain via the vagus nerve. For the healthy group, this process was limited so the accumulation of OSyn only took place in the brain but not in the ENS. For PD patients, this production in the gut might accelerate pathologically, and consequently, the significant increase of OSyn in the colon and brain leads to constipation and mobility disorder.

The dysfunction of the microecology-gut-brain axis plays an important role in the pathogenesis of PD (Tan et al. 2022). Intestinal microecological imbalance increases the risk of PD, which is related to dysbacteriosis, decreased short-chain fatty acids, lipopolysaccharides (LPS), and impaired mucosal barrier function. We found that although the alpha and beta diversity of duodenum and sigmoid mucosal microbiota in PD patients were not significantly different from those in normal individuals, the microflora composition in the duodenal and sigmoid mucosa in PD patients was changed. Specifically, in terms of duodenal mucosal flora, the relative abundances of *Kiloniellales*, *Flavobacteriaceae*, and *CAG56* in healthy controls were higher than those in PD patients, while those of *Proteobacteria*, *Gammaproteobacteria*, *Burkholderiales*, *Burkholderiaceae*, *Oxalobacteraceae*, *Ralstonia*, *Massilla*, and *Lactococcus* in healthy controls were lower than those in PD patients. In terms of sigmoid mucosal flora, the relative abundances of *Thermoactinomycetales* and *Thermoactinomycetaceae* in healthy controls were higher than those in PD patients, while those of *Prevotellaceae* and *B. longum* were lower than those in PD patients.

Previous studies based on fecal flora show that PD patients have intestinal microecological disorders. Zheng et al. (2021) summarized that the relative abundances of *Prevotellaceae*, *Lachnospiraceae*, and *Faecalibacterium* in the feces of patients with PD decreased, while the relative abundances of *Verrucomicrobiaceae*, *Bifidobacteriaceae*, *Christensenellaceae*, and *Ruminococcaceae* increased. In addition, the relative abundances of some opportunistic pathogens including *Porphyromonas*, *Prevotella*, and *Corynebacterium_1* also increased (Zheng et al. 2021). Studies showed that the absolute concentrations of acetate, propionate, and butyrate in fecal samples of PD patients decreased significantly (Unger et al. 2016). Further studies have shown that fecal dysbacteriosis in PD patients is related to the disease course, severity, and clinical manifestations. Specifically, *Lactobacillus gasseri* and *Deferribacterales* were positively correlated with the course of PD, while *Escherichia*, *Shigella*, *Lachnospiraceae*, and *Clostridium coccoides* were negatively correlated with the course of PD. *Enterobacteriaceae*, *Lactobacillaceae*, *Enterococcus*, *Escherichia*, and *Proteus* were positively correlated with the severity of PD disease, while *Lachnospiraceae*, *Blautia*, *Faecalibacterium*, and *Ruminococcus* were negatively correlated with the severity of PD disease. *Enterobacteriaceae* was positively correlated with the severity of PD motor dysfunction, and *Lachnospiraceae* was negatively correlated with PD motor dysfunction (Zheng et al. 2021). Further basic study shows that the signal of intestinal microorganisms is necessary for neuroinflammatory response and antibiotic treatment can improve the pathological changes related to PD in mice. Compared to healthy donors, fecal microbiota transplant (FMT) from PD patients enhanced the pathological damage associated with PD in αSyn overexpressed mice (Sampson et al. 2016). Based on the imbalance of the microecology-gut-brain axis in patients with PD, clinical and basic studies have shown that probiotics (Tamtaji et al. 2019; Sun et al. 2021) and FMT (Xue et al. 2020; Zhao et al. 2021) can protect PD, and its mechanism is related to improving intestinal microecological imbalance, strengthening intestinal mucosal barrier, and reducing inflammation.

The intestinal flora contains three biological layers: the inner layer of bacteria is called membrane flora, binding to specific receptors on the surface of intestinal mucosal epithelium and dominated by obligate anaerobic bacteria such as *Bifidobacterium* and *Lactobacillus*. The middle layer represents facultative anaerobes dominated by *Bacteroides*. The outer layer of bacteria is called luminal flora, attached to the surface of the intestinal mucosa and dominated by *Escherichia coli*, enterococci, and other aerobic or facultative aerobes (Arvans et al. 2005). It can be inferred that the composition of intestinal mucosal flora is different from that of fecal flora, corresponding to the opinion of other scholars (Hou et al. 2022). Keshavarzian et al. (2015) have shown that the relative abundance of *Proteobacteria* in mucosal flora is higher than that in fecal flora. The relative abundance ratio of *Firmicutes* to *Bacteroides* in fecal flora is higher, and the relative abundances of butyrate-producing anti-inflammatory bacteria such as *Blautia*, *Roseburia*, and *Coprococcus* in fecal flora are higher. Besides, the composition of mucosal flora was different in different parts of the gut due to the pH and other environmental factors. Previous studies about dysbacteriosis in PD patients mainly focus on fecal microflora (Qian et al. 2020; Tan et al. 2021). Only sporadic studies have reported sigmoid mucosal flora in PD patients, and there is no report about mucosal flora of the upper digestive tract now. It was found that the change of fecal flora in PD was more prominent than that in sigmoid mucosa. The diversity of sigmoid mucosal microbiota in patients with PD was not different from that in healthy controls, but the composition changed. The relative abundances of *Coprobacillaceae*, *Dorea*, and anti-inflammatory bacteria *Faecalibacterium* in the sigmoid mucosa of PD patients were lower than those of the healthy controls, while the relative abundances of the proinflammatory bacteria including *Oxalobacteraceae* and *Ralstonia* were higher than those of the healthy controls (Keshavarzian et al. 2015).

Similar to the previous studies (Nuzum et al. 2020), this research found that the diversity of mucosal flora in PD patients was the same as that in healthy controls, but the species composition changed significantly. At the phylum level, the relative abundance of *Proteobacteria* in the duodenal mucosa of PD patients increased. *Proteobacteria* includes lots of pathogens, such as *E. coli*, *Salmonella*, *Vibrio cholerae*, *Helicobacter pylori*, and so on (Rizzatti et al. 2017). The relative abundance of *Gammaproteobacteria* in the duodenal mucosa of PD patients increased. The *Gammaproteobacteria* consists of many important pathogens, such as *Salmonella*, *Yersinia*, *V. cholerae*, and *Pseudomonas aeruginosa* (Nina Parker 2016). At the order level, *Burkholderiales*, a species with increased relative abundance in the duodenal mucosa of PD patients, belongs to *Betaproteobacteria*, which also contains some important pathogenic bacteria, such as *Burkholderia* and *Bordetella* (Nina Parker 2016). At the family level, the relative abundances of *Burkholderiaceae* and *Oxalobacteraceae* in the duodenal mucosa of PD patients increased. *Oxalobacterium* belongs to *Burkholderiales* and was isolated from humans and animals’ rumen or the large intestine (Ning et al. 2022). Its high abundance was found to be associated with a high risk of PD (Ning et al. 2022). At the genus level, the relative abundances of *Ralstonia* and *Massilia* in the duodenal mucosa of PD patients increased. *Ralstonia* is a human opportunistic pathogen leading to septicemia, meningitis, and osteomyelitis (Ryan et al. 2014). Although the frequency of human infection is low, the infections are serious (Ryan et al. 2014). *Massilia* is also a pathogenic bacterium, which has been isolated from soil, air, immunocompromised patients, and otitis media patients (Park et al. 2013). It can be seen that the relative abundances of many proinflammatory bacteria in the duodenal mucosa of PD patients increased significantly. However, in this study, the change of microbiota in the sigmoid mucosa of PD patients was not as significant as that in the duodenal mucosa.

Our study applied mIHC for the first time to detect three αSyn species in the intestinal mucosa of PD patients, which was mainly used in the cancer research field. Moreover, we collected normal colonic mucosal tissues in this prospective study. Unlike in previous retrospective studies (Aldecoa et al. 2015; Stokholm et al. 2016), in which only the pathological specimens from the PD lesion sites could be collected, the degraded intestinal tissues were hard to be explored. Our results suggested that intestinal biopsy was helpful for the diagnosis of PD, which might provide a novel strategy for the preclinic PD diagnosis. Additionally, unlike some studies (Qian et al. 2020; Tan et al. 2021) mainly focusing on the fecal flora in PD, this study firstly revealed the characteristics of the mucosal flora in different parts of the digestive tract of PD patients, especially the duodenal mucosa and sigmoid mucosa, helping us fully understand the intestinal microecological disorders of PD patients.

Limitations still exist in this study. Firstly, the number of subjects is limited and more volunteers needed to be recruited to further confirm our results. Secondly, in this study, the patients were not separated into different pathogenesis grades as the sample limitation. Thirdly, this study was observational, so interventional studies are needed in the future, to explore the specific mechanisms.

In conclusion, our study found that the distribution features of three types of α-synuclein, especially the oligomer types, were different between PD patients and healthy controls. The OSyn/αSyn expression in sigmoid mucosa had a potential diagnosis efficiency for PD, making it possible to diagnose PD early by intestinal biopsy. The intestinal mucosal microbiota composition of PD patients altered with the relative abundances of proinflammatory bacteria in the duodenal mucosa increased. The OSyn/αSyn expression level was correlated with mucosal microbiota diversity and composition. This study helps to develop in-depth knowledge of the microecology-gut-brain axis in PD patients, but the specific mechanism still needs to be further studied.

## Supplementary Information

ESM 1: Figures S1–S4 (PDF 6634 kb)

## Data Availability

The data underlying this article are available in the article and supplementary material. Specific data underlying this article are available on request from the corresponding author.
