# Supplementary material for: The alteration of intestinal mucosal α-synuclein expression and mucosal microbiota in Parkinson’s disease
Source: Appl Microbiol Biotechnol. 2023 Feb 16;107(5-6):1917–29. doi: 10.1007/s00253-023-12410-w (PMC10006030; doi:10.1007/s00253-023-12410-w)
Supplement: Supplementary file 1 — Figures S1–S4 (PDF 6634 kb) [file 253_2023_12410_MOESM1_ESM.pdf]

APPLIED MICROBIOLOGY AND BIOTECHNOLOGY

Supplementary materials for
The alteration of intestinal mucosal α-synuclein expression and mucosal microbiota in Parkinson's disease

Jihua Shi [1], Yiran Wang [1,2], Dan Chen [1], Xue Xu [1], Wenbin Li [1], Kai Li [3], Jing He [3], Wen Su [3], Qingfeng Luo [1*]

1. Gastroenterology Department, Beijing Hospital, National Center of Gerontology; Institute of Geriatric Medicine, Chinese Academy of Medical Science, Beijing, China
2. Peking University Fifth School of Clinical Medicine, Beijing, China
3. Neurology Department, Beijing Hospital, National Center of Gerontology; Institute of Geriatric Medicine, Chinese Academy of Medical Science, Beijing, China

* Corresponding author:

Qingfeng Luo, Telephone number: 13811519095, Email address: luoqf2000@126.com

Postal address: Gastroenterology Department, Beijing hospital, No.1 Dahua Road, Dongdan, Dongcheng District, Beijing, China, 100730

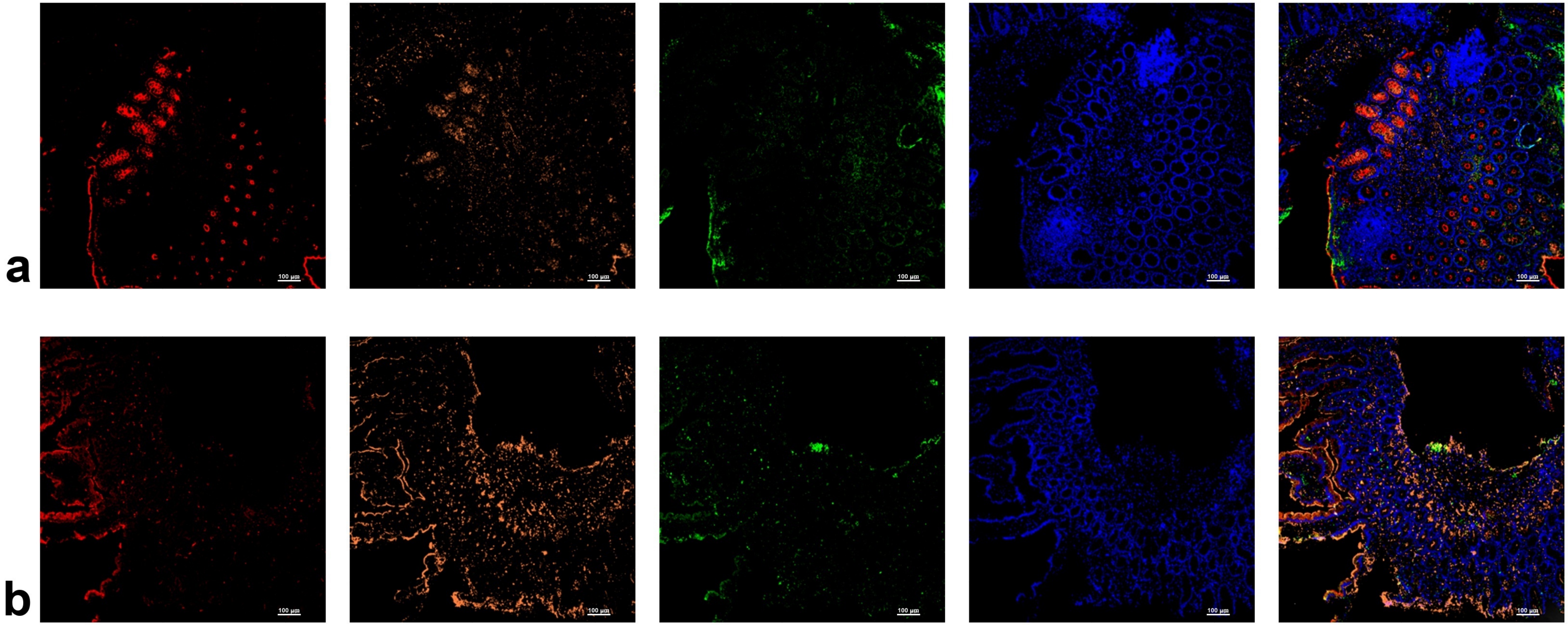

Figure S1 The distribution of different types of aSyn in duodenal mucosa of healthy control (a) and PD patients (b); ×100. The scale bars are in the bottom right corner of each picture, indicating 100 μm. As listed from left to right: red is total α-synuclein; orange is oligomer α-synuclein; green is phosphorylate α-synuclein; blue is cell nucleus; multi-color is the merged picture.

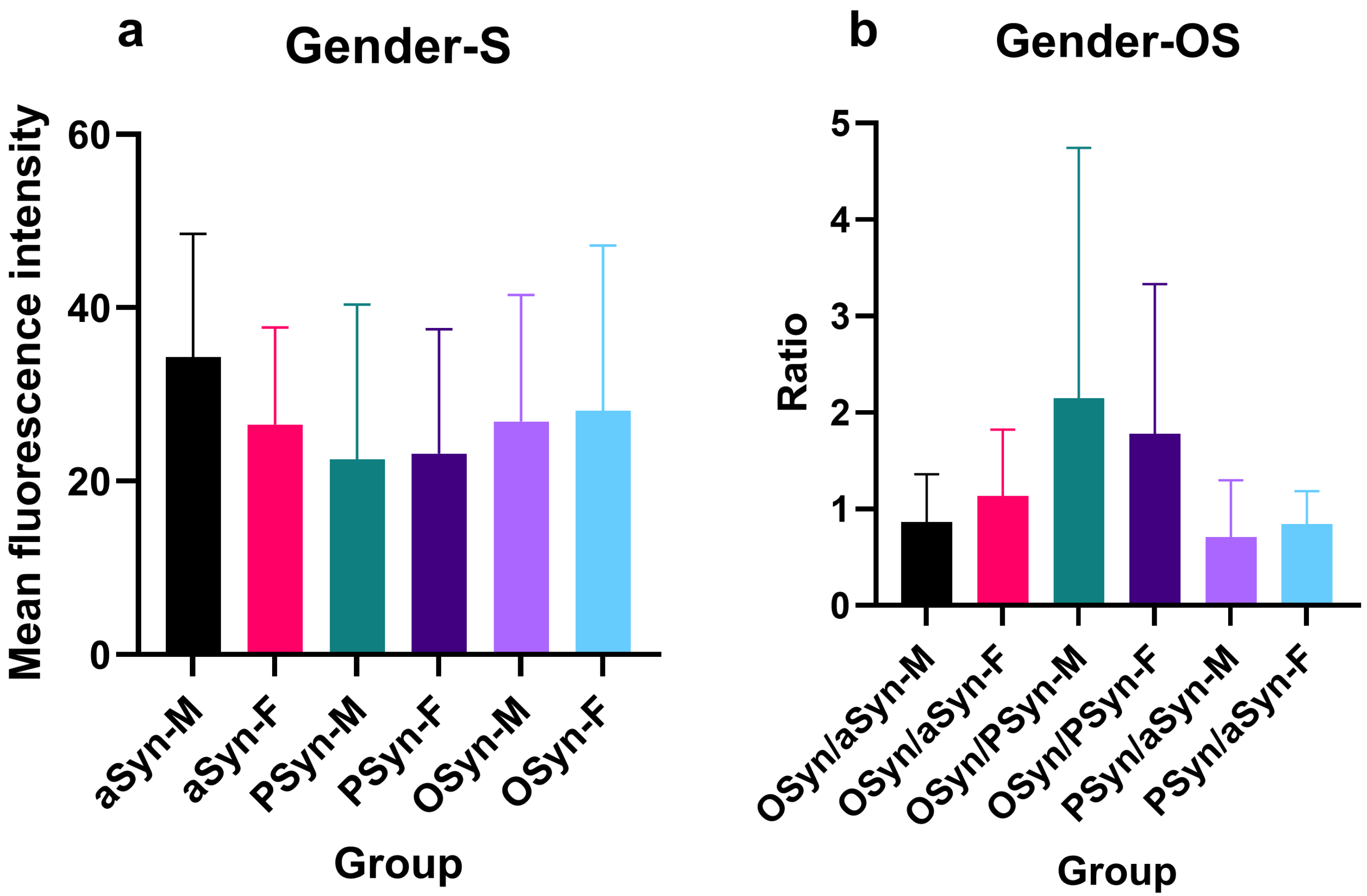

Figure S2 The mean fluorescence intensity (a) and ratio (b) of different types of α-synuclein are grouped by sex. M: male; F: female. aSyn: total α-synuclein; OSyn: oligomer α-synuclein; PSyn: phosphorylate α-synuclein.

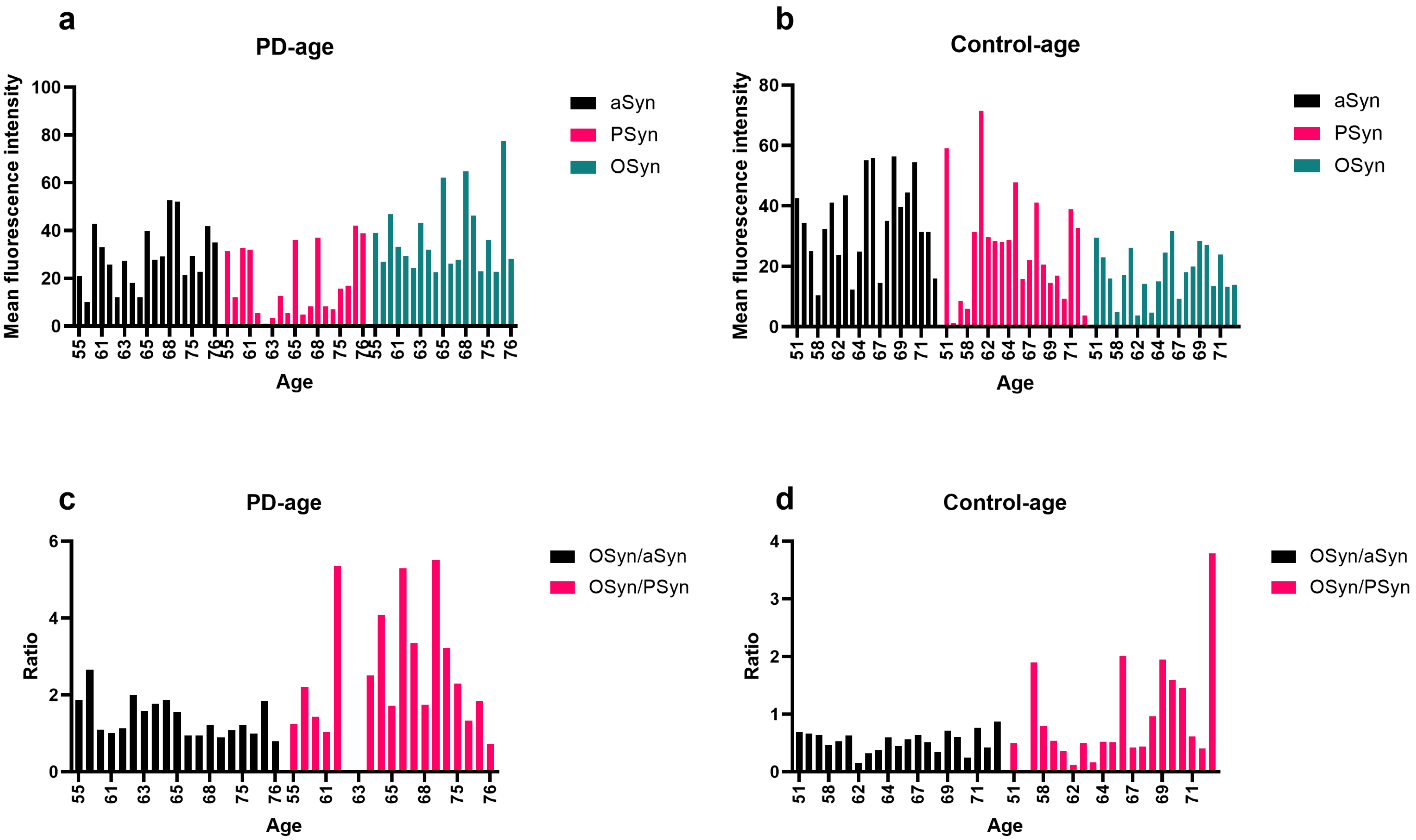

Figure S3 The mean fluorescence intensity (a) and ratio (b) of different types of α-synuclein showed according to age. aSyn: total α-synuclein; OSyn: oligomer α-synuclein; PSyn: phosphorylate α-synuclein.

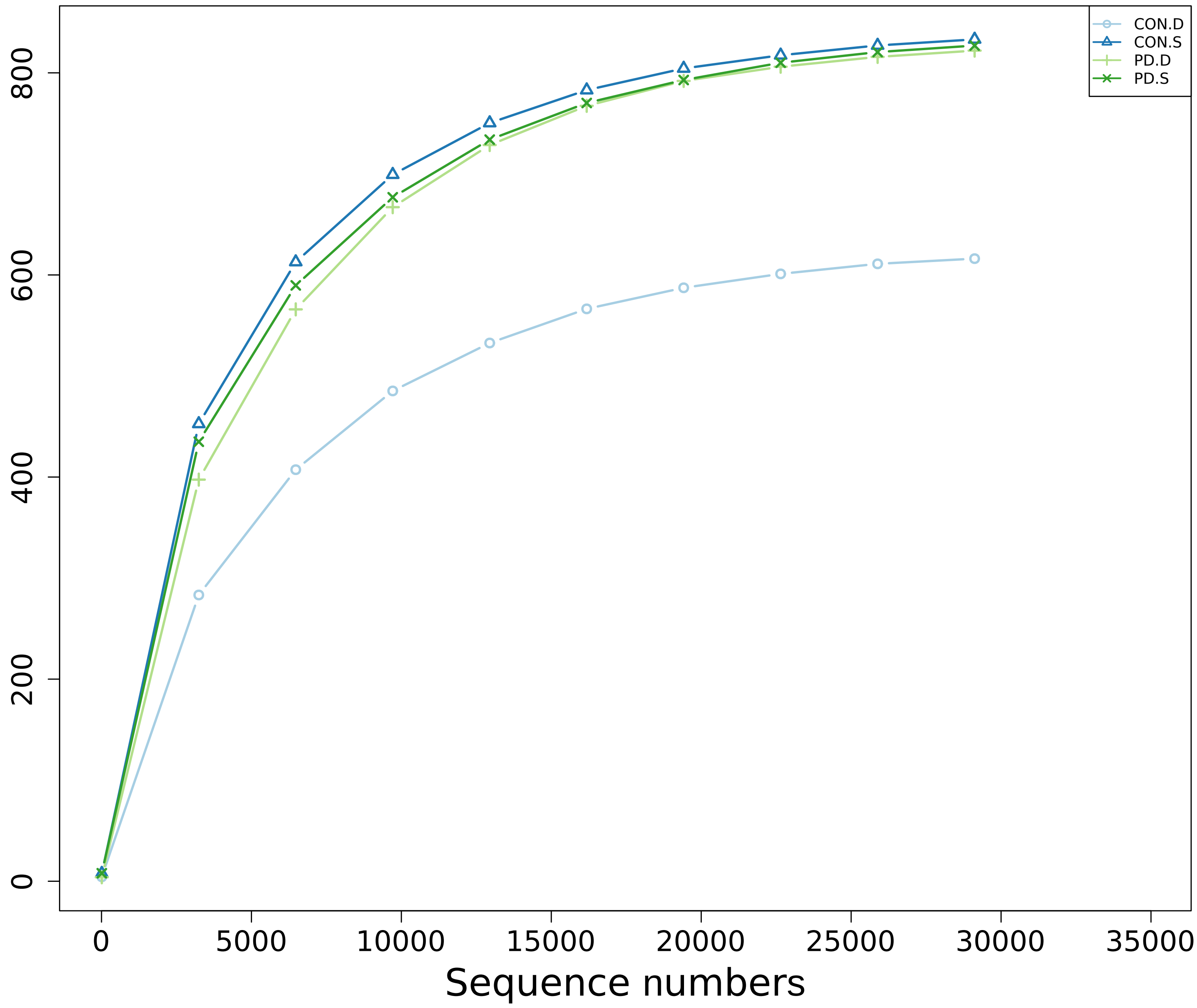

Figure S4 Sample rarefaction curves for observed species per group.
